# Septic arthritis of both knees following intra-articular injection of petrol

**DOI:** 10.5249/jivr.v7i1.341

**Published:** 2015-01

**Authors:** Alireza Janbakhsh, Feizollah Mansouri, Siavash Vaziri, Babak Sayad, Mandana Afsharian, Parviz Ghaffari

**Affiliations:** ^*a*^Department of Infectious Disease, Imam Reza Hospital, Kermanshah, Iran.; ^*b*^Department of Orthopaedic Surgery, Imam Reza Hospital, Kermanshah, Iran.

**Keywords:** Septic arthritis, Petrol, Intra-articular, Injection

## Abstract

A 70 years old man was referred to our center with bilateral knee arthritis following intra-articular petrol injection. Because of previous antibiotics use gram stain and culture were negative. Septic arthritis was diagnosed and antibiotics and drainage were started. After 2 years he improved eventually and was able to walk. But, some movement limitation remained.

## Introduction

Septic arthritis is a joint infection with various bacterial, fungal and mycobacterial causes. Its annual prevalence is estimated at 2-10 /100,000, increasing due to greater predisposing factors and operation rates in recent years. It is believed that bacterial arthritis usually evolves during both overt and occult bacteremia including endocarditis. Therefore, native, diseased or prosthetic joints are at risk.^1^ Predisposing factors for septic arthritis include age older than 80 years, diabetes mellitus, rheumatoid arthritis (RA), prosthetic joint, joint surgery, skin infection and ulcer, IVDU, alcoholism, and intra-articular injection of steroid. Since in many cases predisposing factors are not explicitly identifiable, septic arthritis should be considered as a probable diagnosis in every joint inflammation.^2^

Intra-articular injection of drugs for treatment of inflammatory and non-inflammatory joint disease can result in septic arthritis. Yet, there is no report with respect to injection of petrol as a cause of septic arthritis in the literature. In this study we report the first case of petrol-induced knee septic arthritis. 

## Case presentation

A 70 years old man was referred to a private hospital in December 2010 due to bilateral pain and swelling in both knees that lasted 10 days after receiving petrol injection for treatment of degenerative joint disease (DJD). He complained of swelling, pain and redness, which appeared in both knee joints a day after the injection. Subsequently he received drainage and irrigation on both knees in a hospital in Koohdasht (a small city near Kermanshah, a western province in Iran). Cefazolin and gentamicin also were prescribed. 

**Figure 1:**
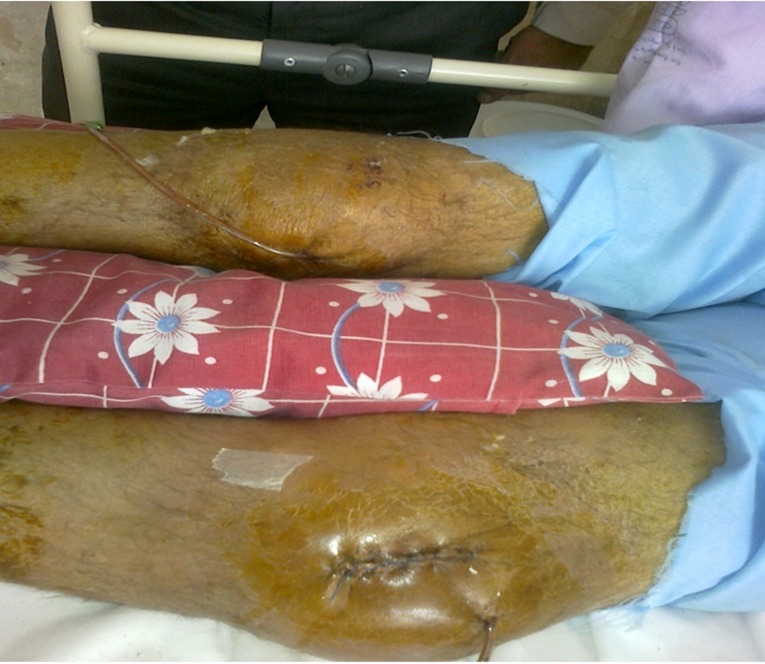
Shows patient knee in second day of admission.

After a few days, he was referred to a private hospital in Kermanshah, while suffering from fever of 39 degrees centigrade (102.2 degrees Fahrenheit). Consequently Teicoplanin, Clindamycin and Imipenem were prescribed. Lab analysis showed WBC=16100, Hb= 7.6 g/dl, CRP=+++ and ESR= 105mm/h. while other lab tests were normal. Furthermore, synovial fluid aspiration was purulent and showed WBC=49290 (PMN=90%, Lymph=10%), Pr= 1.9 g/L, LDH=1586 and sugar= 53mg/dl and gram stain, was negative. In addition doppler sonography was normal. (Figure 1)

After receiving peer consultations, patient was operated once again and drainage and joints irrigation were performed, thus, 500 milliliter pus was drained from one knee and the result of the subsequent culture test turned out negative. Four units of packed cell were transfused and gradually fever and joint inflammation subsided (i.e., in 5 days), ESR decreased to 37mm/h and WBC was 5000/ml.

**Figure 2:**
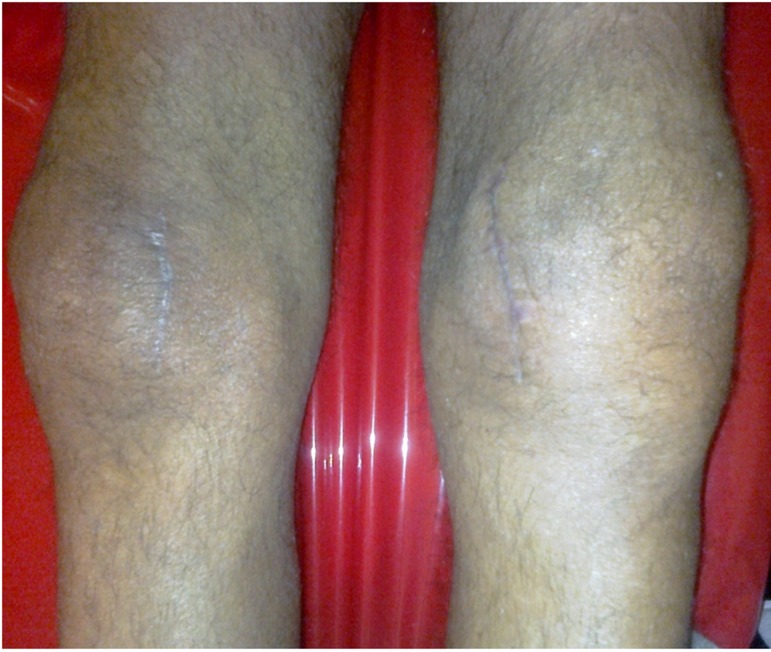
Shows patient knee joint at last days at Imam Reza hospital.

 The patient, then, was referred to a university hospital. Teicoplanin, Clindamycin and Imipenem were replaced with Ciprofloxacin and clindamycin. Joints were dry tap at this time. During the admission patient was well and had no fever. Pain and other inflammation signs were resolved considerably. (Figure 2)

Twenty eight days after his first admission, patient was discharged from hospital and oral Ciprofloxacin and Clindamycin were continued for additional two weeks. At this time patient was able to stand up and walk slowly, and in general he was well. During the following two-post discharge visits his condition was stable and he only complained of minor movement limitations and pain. Two years post-discharge, the patient was able to walk with crutch and only had a few symptoms of DJD.

## Discussion

Iatrogenic septic arthritis can develop as a result of joint therapy and diagnostic procedures such as arthroscopy, surgery, and intra-articular drug injections. The prevalence of septic arthritis is increasing. In Iceland, between 1990 and 2002, it increased from 2.2 to 9 cases per year. ^3^ The latest studies show that joint infection following arthroscopy is rare and typically appears 8 days after the procedure. ^4^ Coronary angiography is a known rare cause of hip septic arthritis. ^5^ However, septic arthritis is more common post steroid intra-articular injection ^6,7^ and can cause death if undiagnosed or diagnosed late.^8^

Osteoarthritis is one of the most common causes of joint pain resulting in damages to the cartilage overlying join surface. With time, if the wear becomes severe, it could expose the underlying bone. Some factors including tendon and cartilage damage, fracture, obesity and genetic tendency may also result in DJD. Invasive treatments such as corticosteroid and hyaluronic acid ^9^ and osmic acid, which have been used for treating DJD, may result in septic arthritis.^10^ Although, in some cases, petrol injection is used for suicide, para-suicide and self-deliberate harms, but its use for treatment of DJD and related septic arthritis has not been reported. This report is the first case of petrol-induced septic arthritis one day post injection. Petrol is a chemical material that can cause damage to any tissue and organ because it can produce severe necrosis and infection following injection to body tissues.

In the current case, we could not identify any etiologic agent that could have been related to previous antibiotic use. Staphylococcus aureus is known as the leading cause of septic arthritis in RA, IVDU, and cases without predisposing factors followed by Pseudomonas aeruginosa, Streptococcus pyogenes, Haemophilus influenza,^1^ however, other agents including Ureaplasma urealyticum, Salmonella, Listeria monocytogenes,^11^ Mycobacterium abcessus^12^ have been reported as etiologic agents of iatrogenic septic arthritis.

The prescription of antibiotics led to a favorable treatment outcome in our patient, therefore, we believe that Staphylococcus aureus or gram negative rods could have been the causative agent. Ultimately, use of antibiotic treatment and drainage, in two steps, improved the condition of the patient and he was able to walk using crutch.
